# Clinical Impact of the Administration of FOLFIRINOX Beyond Six Months in Advanced Pancreatic Adenocarcinoma: A Cohort Study

**DOI:** 10.7759/cureus.19361

**Published:** 2021-11-08

**Authors:** Cornelia Nitipir, Radu Vrabie, Andreea Ioana Parosanu, Raluca Tulin, Bogdan Cretu, Adrian Cursaru, Iulian Slavu, Adrian Miron, Valentin Calu, Maria Cristina Orlov Slavu

**Affiliations:** 1 Oncology, Elias Emergency University Hospital, Bucharest, ROU; 2 Oncology, Carol Davila University of Medicine and Pharmacy, Bucharest, ROU; 3 Endocrinology, Emergency Hospital Prof. Dr. Agrippa Ionescu, Bucharest, ROU; 4 Orthopedics and Traumatology, University Emergency Hospital Bucharest, Bucharest, ROU; 5 Orthopedics, Carol Davila University of Medicine and Pharmacy, Bucharest, ROU; 6 Surgery, Emergency Hospital Prof. Dr. Agrippa Ionescu, Bucharest, ROU; 7 Surgery, Elias Emergency University Hospital, Bucharest, ROU

**Keywords:** survival, maintenance, dose-intensity, pancreatic adenocarcinoma, chemotherapy

## Abstract

Background

Although a toxic regimen, FOLFIRINOX is one of the most efficient chemotherapy regimens in advanced pancreatic adenocarcinoma. There is no standard number of cycles in locally advanced or metastatic stages.

Materials and method

The present retrospective study reports the experience of a single center with this regimen administered until disease progression or unacceptable toxicity. The authors of this retrospective study analyzed the data on patients with this diagnosis treated in our clinic during 2017-2021. Forty-two patients were included in the study, 21 who received six courses or less and 21 who received more than six courses. Progression-free survival (PFS) and overall survival (OS) were analyzed according to this stratification. The oncological response was also reported according to dose reduction and treatment delay, irrespective of the number of courses administered.

Results

Median PFS was 7.5 months, and median OS was 13.6 months in the entire studied population. When patients were compared according to the number of courses received (under six vs. over six), there were obvious differences (PFS: 5.17 months vs. 11.2, p = 0.8, OS: 8 months vs. 17.3 months, p = 0.6). However, when stratifying survival by treatment delay and the presence or absence of dose reduction, better results were seen with lower doses (p<0.001) and treatment temporization (p=0.03). The general incidence of hematologic and neurologic toxicity was higher than the ones reported in the literature.

Conclusion

The study revealed that patients benefit from the administration of FOLFIRINOX for more than six months, but that the administration of full dose and the maintaining dose intensity does not necessarily favor the patient.

## Introduction

Pancreatic cancer is a disease with high mortality, causing over 400,000 deaths annually [1], with median survival in the advanced stages of about 12 months and for the metastatic stage of about six months. At the time of diagnosis, 15% are diagnosed in resectable stage (I and II), 35% in locally advanced stage, and over 50% are in metastatic stage [2]. FOLFIRINOX is a reference regimen extremely effective in advanced pancreatic adenocarcinoma. Better survival with this type of chemotherapy, when compared with gemcitabine monotherapy, has been demonstrated in the pivotal PRODIGE trial (11.1 vs. 6.8 months, hazard ratio (HR) for death 0.57; 95% confidence interval (CI); p <.0001) [3]. However, the regimen also involves significant toxicity, which is why lower dose administration has been proposed. Despite these proposals, the doses that maintain the optimal toxicity-benefit ratio are unknown [4,5]. The number of treatments required for FOLFIRINOX is not standardized, with the option of administering this regimen until unacceptable toxicity or progression to maintain the oncological benefit, but also the option of maintenance treatments or even follow-up of patients who have obtained disease control to limit toxicities [6]. The present study reports experience with this regimen administered up to unacceptable toxicity or disease progression, with a follow-up period of four years, quantifying the benefit of survival, but also the impact of maintaining dose intensity on the patients’ benefit.

## Materials and methods

The present study is retrospective, observational, monocentric and included all histologically confirmed patients with locally advanced or metastatic pancreatic cancer, treated in the first line in the Oncology Clinic of Elias University Emergency Hospital with FOLFIRINOX. The study included patients who presented between 2017 and 2020 and follow-up time was between 2017-2021. Data were collected with the approval of the Institutional Ethics Committee of Elias Emergency University Hospital, approval number 7164/13.10.2021.

All patients received this regimen according to our clinic-specific protocol (which also includes supportive medication for primary prophylaxis of possible toxicities). Chemotherapy was continued until disease progression or unacceptable toxicity. None of the patients who received less than six courses stopped treatment due to disease progression (most did so due to unacceptable toxicity or personal decision). If an interval longer than three weeks between courses was present, the patients were not included in the present analysis.

Our data sources were patients' medical records and histories, and a collection was made, keeping the subjects anonymous. These included the particular features of the patients (gender, age, performance status), the number of courses administered, details on the degree of toxicity (according to National Cancer Information Center common terminology criteria for adverse events [NCIC-CTCAE]) [7], the time from the beginning of the administration to the last dose, treatment delay, the proportion of dose adjustment for each chemotherapy agent, but also data on the antitumor response. Progression-free survival (PFS) and overall survival (OS) were measured.

Delayed treatment and dose reduction were the most important parameters in evaluating the oncological response, irrespective of the number of courses administered.

Technical details on administration

All enrolled patients began with full doses of chemotherapy. A course of chemotherapy involved the administration of oxaliplatin 85 mg / m2, irinotecan 180 mg / m2, leucovorin 400 mg / m2, fluorouracil 400 mg / m2 bolus, followed by fluorouracil 1200 mg / m2 continuous infusion over 46 hours. Supportive medication administered during the course included: hydration with saline solution, Ringer's solution and 5% glucose solution, histamine H2 receptor blockers, dexamethasone, serotonin 5HT-3 receptor antagonists (granisetron), atropine, and if needed: loperamide and furosemide. The regimen has been administered to all patients under close medical supervision. Verification of the interaction with concomitant medication of the patient was performed at each visit. The collaboration with the neurology service, which is very familiar with the pathology related to the oncology treatment, allowed the identification of early oxaliplatin-induced neuropathy and the adjustment of the doses accordingly. According to the clinics' protocol, in case of toxicity grade two or higher, the treatment was delayed until adverse events remission. If neurological grade three toxicity occurred, oxaliplatin treatment was permanently discontinued.

Statistical analysis

IBM SPSS Statistics v.20 (IBM Inc., Armonk, New York) was used for data processing. The Pearson Ki2-test was used to compare patient characteristics, including toxicity. The Kaplan-Meier method was used to compare PFS (progression-free survival) and OS (overall survival) according to the number of treatment courses and other proposed features. PFS was defined as the time in months between the first administration until the radiological progression according to RECIST 1.1 criteria [8]. OS was defined as the time in months from first administration to death. The value of P <.05 was considered statistically significant and was calculated using the log-rank test.

## Results

A total of 42 patients were enrolled in the present trial, of which 21 received at least six courses of chemotherapy and 21 received over six courses. Patient characteristics are listed in Table 1.

**Table 1:** Patient characteristics stratified by the number of FOLFIRINOX courses received ECOG - Eastern Cooperative Oncology Group

Characteristic	All patients N=42	Six FOLFIRINOX courses or less	More than six FOLFIRINOX courses
Gender			
Female	13	5	8
Male	29	16	13
Age			
Median	62	67	57
Std. dev	10.3	11.2	12.6
Range	(37-80)	(40-80)	(37-68)
ECOG performance status			
ECOG=1	20	19	1
ECOG=0	22	2	20
Tumor location (%)			
Head	30 (71.4%)		
Body	9 (21.4%)		
Tail	2 (4.8%)		
Patients with treatment delay (%)	34 (81%)	18	16
Patients with dose reduction	18 (42.9%)	10	8

The average number of FOLFIRINOX courses was 9.5 (2-25, std dev 6.3), the total number being 399. The maximum duration of treatment was one year and five months, and the minimum was one month with a median of 8.5 months.

57.1% received the full dose throughout treatment. 17.07% of patients had grade 3-4 toxicity. The most common toxicities were sensory and hematological neuropathy of any degree (anemia 17.07%, thrombocytopenia 19.51%, neutropenia 34.14%). Sensory neuropathy occurred in 34 patients, eight of them with grade one (23.5%) toxicity, 20 of them with grade two (58.8%), and six with grade three (17.7%). Thirty-four patients (82.92%) had delays in treatment related to toxicity.

Data related to the oncological result of the treatment and stratification on the number of cures received are summarized in Table 2.

**Table 2:** Oncology efficacy endpoints stratified by the number of FOLFIRINOX courses received PFS - progression-free survival, OS - overall survival

Oncology efficacy endpoints	All patients (N=42)	Six FOLFIRINOX courses or less	More than six FOLFIRINOX courses	P-value
Stable disease	2	1	1	
Partial response	3	1	2	
Complete response	0	0	0	
Progressive disease	37	19	18	
PFS months median	7.5	5.17	11.2	.08
(range)		(1-13)	(3-35)	
OS months median	13.6	8	17.3	.06
(range)		(4-9)	(5-42)	

**Figure 1:**
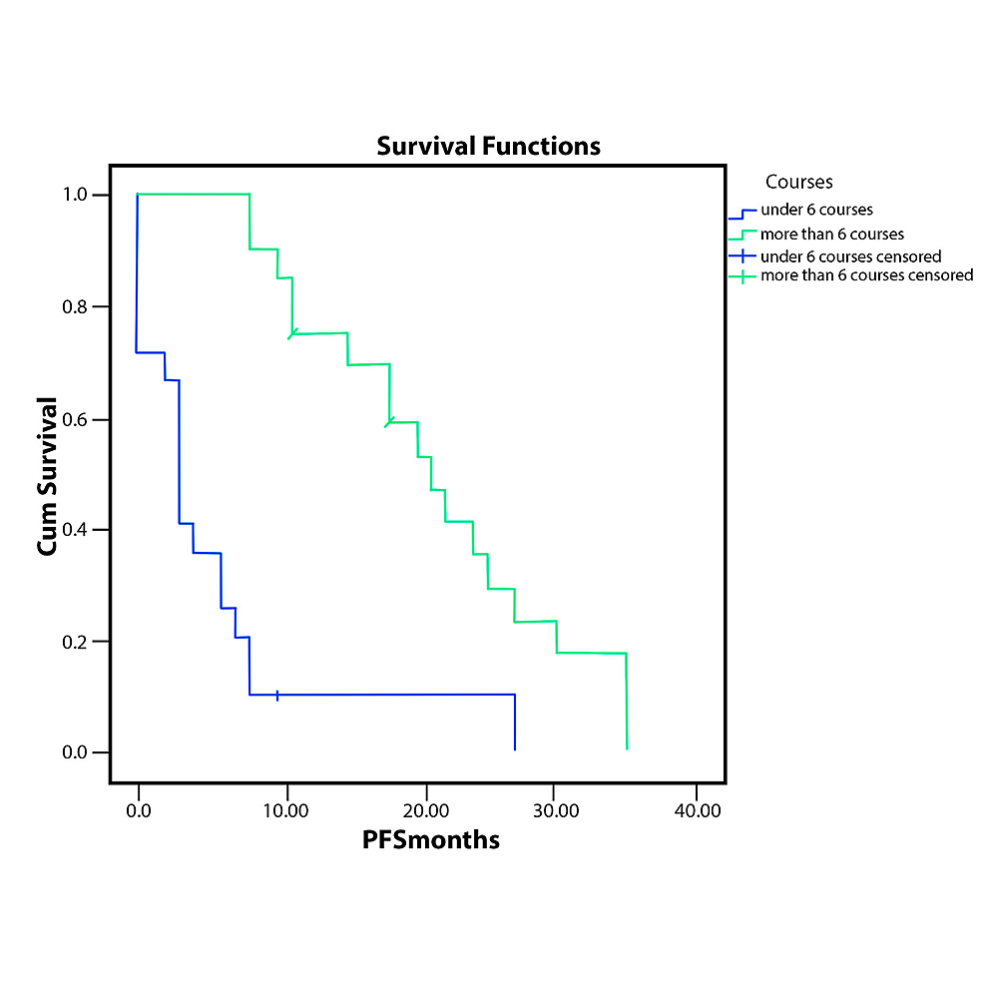
PFS according to the number of FOLFIRINOX courses received (p<.001 PFS - progression-free survival

**Figure 2:**
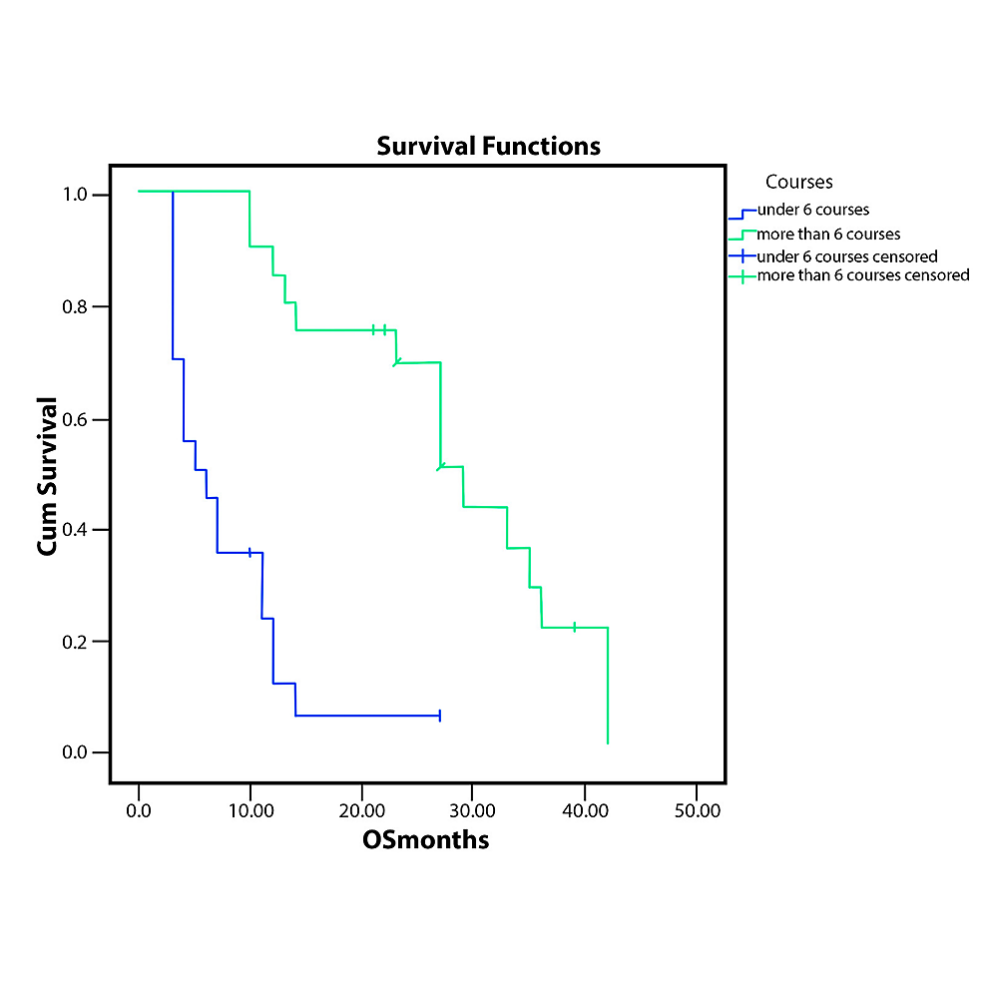
OS according to the number of FOLFIRINOX courses received (p<.001 OS - overall survival

Patients who received more than six courses had better PFS and OS (p<.0001) for each case, as Figure 1 and Figure 2 show.

**Figure 3:**
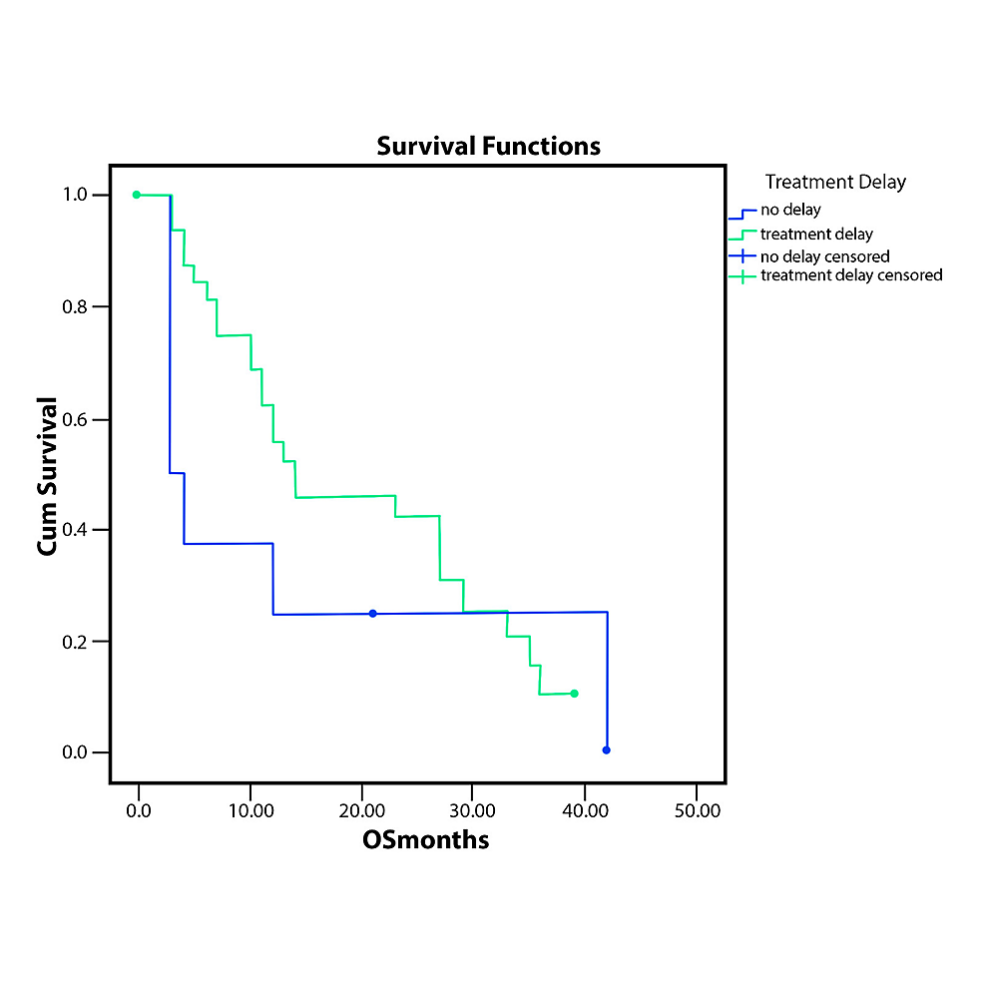
OS stratified by the presence or absence of treatment delay (p=0.33) OS - overall survival

**Figure 4:**
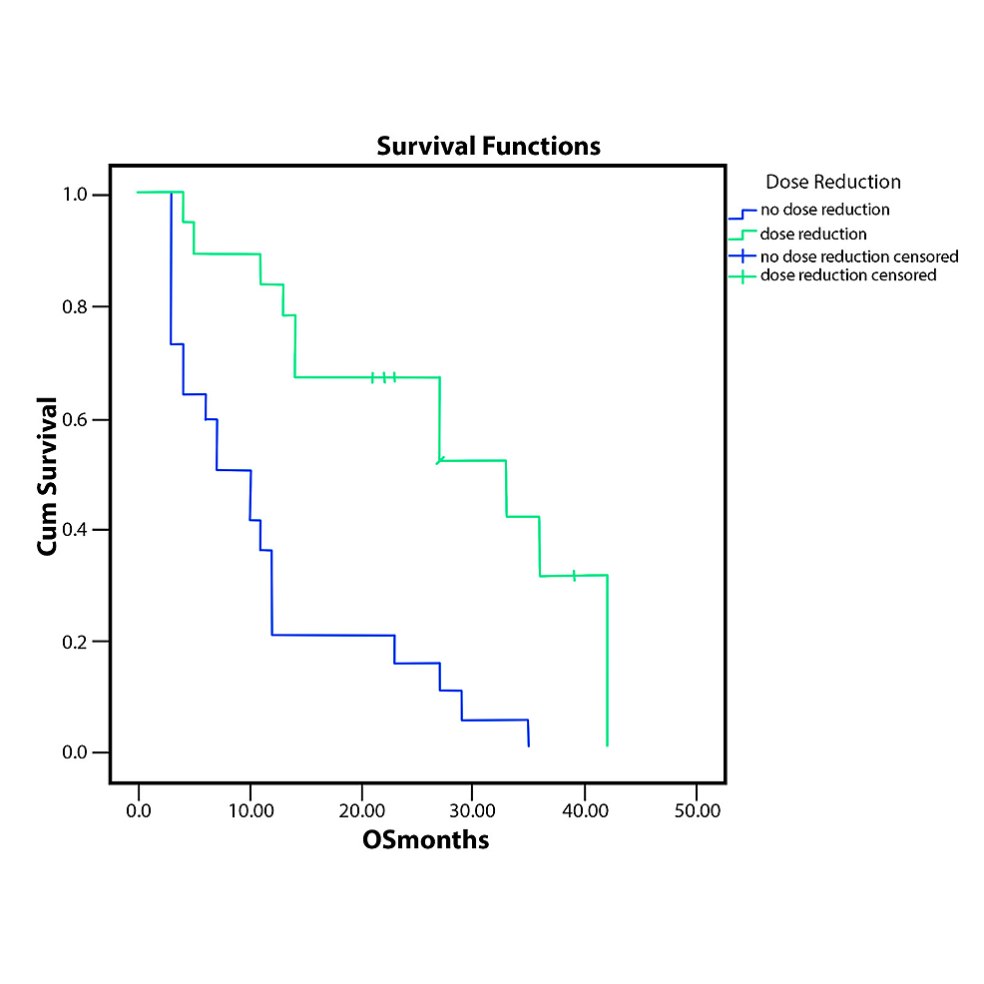
OS stratified by the presence or absence of dose reduction (p<.0001 OS - overall survival

Patients with treatment delay had better overall survival than the ones without and so had the ones with dose reduction. The analysis was made without any other stratification. The results are represented in Figure 3 and Figure 4.

## Discussion

In the study that established FOLFIRINOX as first-line treatment in patients with advanced pancreatic cancer, the treatment was given for six months to those who had a stable disease or oncological response [3].

In another study meant to compare the toxicities between mFOLFIRINOX (modified with lower doses of irinotecan and no bolus infusion of 5-fluorouracil) and FOLFIRINOX, administration at more than six months for metastatic patients and at more than four months for patients with advanced disease, treatment was possible until disease progression or unacceptable toxicity. However, toxicity data were only reported for standard durations [9].

In the long-term treatment of advanced pancreatic adenocarcinoma, there is also the option of maintenance treatments. Data on the effectiveness of this alternative are scarce and often come from retrospective studies. In one French study, after four or six months of FOLFIRINOX, patients with metastatic pancreatic adenocarcinoma were treated with maintenance capecitabine (2500 mg / m2, d1-14, qd22). The median time to progression was five months under maintenance treatment, and the most common adverse reaction reported was hand-foot syndrome [10].

In the PRODIGE 35 / PANOPTIMOX phase 2 trial, 91 patients (arm A) received maintenance therapy with 5-fluorouracil / leucovorin (leucovorin 400 mg / m2 day 1, 5-fluorouracil 400mg / m2 bolus, 5-fluorouracil 2400mg / m2 46h; q14d). This group was compared with arm B (n = 92 patients) who remained under observation after 4-6 months of FOLFIRINOX, and this treatment was readministered after progression. The median time to progression was 3.3 months in arm A. Due to this short duration, the early reintroduction of FOLFIRINOX was required in the vast majority of patients, leading to a higher total dose of oxaliplatin and an increased frequency of neurological toxicity [11].

Data have also been published on FOLFIRI in maintenance (omission of oxaliplatin after six months makes sense in limiting sensory neuropathy, one of the most common toxicities due to continuation of treatment). A retrospective trial by Caspar et al. reports an average time to progression of eight months, the longest interval with cytotoxic chemotherapy in maintenance [12].

There is also the option of maintenance treatment with olaparib in patients with germline mutations of breast cancer gene 1 or 2 (BRCA1 or 2) who have responded to cancer treatment, as demonstrated by the POLO trial. It is known, of course, that patients with BRCA mutations are more prone to respond to platinum-containing regimens, and 85% of those who received the poly adenosine diphosphate-ribose polymerase (PARP) inhibitor received FOLFIRINOX. We can speculate that this created bias. Progression-free survival was improved in the intention to treat the population (7.4 vs. 3.8 months), but there was no benefit in overall survival [13].

The progression-free survival reported in the literature for patients with metastatic pancreatic adenocarcinoma with FOLFIRINOX is six months, and the overall survival is approximately 11 months [3,14-17]. For the entire population studied in the present study, similar values ​​were obtained (PFS = 7.5 months, OS = 13.6 months). When patients were compared according to the number of courses received (under six versus over 6), there were obvious differences (PFS: 5.17 months vs. 11.2, p = 0.8, OS: 8 months vs. 17.3 months, p = 0.6).

In the PRODIGE study, the most common toxicity was neutropenia (present in 45.7% of patients, in any degree), with only 5.4% having grade four neutropenia [3]. There was also one death caused by treatment, also in the context of febrile neutropenia. Hematologic toxicity was also the most common in our study, with a higher percentage of grade 3-4 neutropenia. However, no deaths were reported in this context, probably due to the smaller number of patients studied. The rates of thrombocytopenia and anemia were also higher in our study (19.5% vs. 9.1% and 17.07% vs. 7.9%). Sensory neuropathy occurred in 9% of PRODIGE patients, regardless of grade [3]. The much higher rate of sensory neuropathy in our study is, of course, explained by the much longer exposure to oxaliplatin.

The success in administrating toxic regimens like FOLFIRINOX for a long time lies in some basic principles used in our clinic. Among them, the most important are: prevention of cold exposure, especially of the extremities, which would lead to a faster onset of neuropathy and worsening of symptoms, an active lifestyle with daily exercise, and walking for at least one hour a day. Also, adherence to the schedule of administration of neurotoxic drugs is extremely important (like the case of oxaliplatin to prevent early neurological damage, including pharyngolaryngeal dysesthesia). In patients at risk of basic neuropathic involvement, for example, in those with diabetes, a neurological evaluation with electromyography is useful at the beginning of treatment and then at short-term follow-up. Prevention of hydro-electrolytic imbalances is held in mind with every chemotherapy course, as well as the restraint in the consumption of neurotoxic substances such as alcohol or tobacco. We also encourage a diversified diet, including substances rich in neurotrophic vitamins such as seeds, fruits, vegetables, fish, caviar, mushrooms.

The limitations of the present study consist in its' retrospective nature and the relatively small number of patients involved. With a higher number of patients, the implication of dose reduction and treatment delay could have been reported for each subgroup (the patients who received six or fewer chemotherapy cycles versus the ones who received more than six, this adding value to the results.

## Conclusions

To conclude, administration of FOLFIRINOX until unacceptable toxicity or disease progression is superior to six-month-only administration. However, it seems that the absence of treatment delay and maintaining the full dose are not factors that contribute to better survival, quite the opposite. This may be an argument for choosing a milder maintenance regimen, such as those suggested by the literature. The toxicity rate was, as expected, higher in the studied population, and this also has to be weighed against the oncological benefit. A prospective trial is needed in order to better analyze these conclusions.
